# Ascorbate protects liver from metabolic disorder through inhibition of lipogenesis and suppressor of cytokine signaling 3 (SOCS3)

**DOI:** 10.1186/s12986-020-0431-y

**Published:** 2020-03-04

**Authors:** Yingying Xu, Yuhang Wu, Yue Xiong, Jiawang Tao, Tingcai Pan, Shenglin Tan, Ge Gao, Yan Chen, Nasir Abbas, Anteneh Getachew, Yuanqi Zhuang, Kai You, Fan Yang, Yin-xiong Li

**Affiliations:** 1 Institute of Public Health, Guangzhou Institutes of Biomedicine and Health (GIBH), Chinese Academy of Sciences (CAS), Guangzhou, 510000 China; 2 Guangdong Provincial Key Laboratory of Biocomputing, GIBH, CAS, Guangzhou, 510000 China; 3 University of Chinese Academy of Sciences, Beijing, 100000 China; 4 Key Laboratory of Regenerative Biology, South China Institute for Stem Cell Biology and Regenerative Medicine, GIBH, CAS, Guangzhou, 510000 China; 5 Guangdong Provincial Key Laboratory of Stem Cell and Regenerative Medicine, Guangzhou, China

**Keywords:** Guinea pig, Lipidomics, Lipogenesis, Insulin signaling, STAT3

## Abstract

**Background:**

Fatty liver is a reversible status, but also an origin stage to develop to other metabolic syndromes, such as diabetes and heart disease that threatens public health worldwide. Ascorbate deficiency is reported to be correlated with increasing risks for metabolic syndromes, but whether ascorbate has a therapeutic effect is unknown. Here, we investigated if ascorbate treatment alone could work on protecting from the development of steatosis and mechanisms beyond.

**Methods:**

Guinea pigs were fed with a chow diet or a high palm oil diet (HPD) respectively. HPD induced animals were administered different concentrations of ascorbate in different time intervals through water. Besides, hepatocyte-like cells derived from human embryonic stem cells and HepG2 cells were treated with palmitic acid (PA) to induce lipid accumulation for molecular mechanism study.

**Results:**

We find that ascorbate rescues HPD and PA induced steatosis and insulin tolerance in vivo and in vitro. We demonstrate that ascorbate changes cellular lipid profiles via inhibits lipogenesis, and inhibits the expression of *SOCS3* via STAT3, thus enhances insulin signal transduction. Overexpression of *SOCS3* abolishes the ascorbate rescue effects on insulin signal and lipid accumulation in hepatic cells.

**Conclusions:**

Ascorbate ameliorates hepatic steatosis and improves insulin sensitivity through inhibiting lipogenesis and *SOCS3*.

## Introduction

Metabolic syndrome has become an epidemic case with detrimental effects on public health and is thought to be an origin of many other metabolic diseases. From the known metabolic syndromes, hepatic steatosis and insulin resistance, which are closely related to development of non-alcohol fatty liver disease (NAFLD) and type 2 diabetes mellitus (T2D), are in association with ectopic accumulation [1].

Insulin resistance is a pathological condition in which cells fail to respond to insulin normally. Excess lipid metabolites trigger a number of different serine kinases with subsequent impairments of insulin signaling or prevent activation of Akt2 [2–4], resulting in insulin resistance. Insulin resistance increases releasing of fatty acids from adipose and delivery of glucose as well as fatty acids to liver, inducing de novo lipogenesis and fatty acid esterification in hepatocytes, resulting ectopic lipid accumulation and steatosis [5]. SOCS3, which is a negative regulator of the JAK/STAT pathway is implicated in hypertriglyceridemia associated with insulin resistance and leptin resistance [6, 7]. Thus, inhibition of SOCS3 is believed to play a central role in metabolic syndrome [8].

Ascorbate is an essential element of organisms due to its capacity of anti-oxidant, anti-inflammation and therapeutic effects for many diseases. It is a coenzyme of many enzymes, including 7α-hydroxylase, which catalyzes the conversion of cholesterol to 7α-hydroxycholesterol [9]. In addition, it is a well-known anti-oxidant, which reduces the content of reactive oxygen species in hepatocyte mitochondria. For a long time, many researches have shown that ascorbate deficiency is correlated to increasing risks for NAFLD and atherosclerosis according to animal models and epidemiological surveys [10–13]. However, further investigations on ascorbate intervention in disease progression have been reporting inconsistent outcomes on animal and human studies [14–17]. Here, we have investigated if ascorbate treatment alone could be of therapeutic value to protect the development of steatosis and mechanisms more than anti-oxidation.

We found that ascorbate ameliorated hepatic steatosis induced by excess saturated fatty acid influx through changing cellular lipid profiles as well as improving insulin sensitivity which was related to inhibition of *SOCS3*.

## Method

### Cell culture

Cells were maintained at 37 °C with humidified air and 5% CO_2_. HepG2 cells were cultured in DMEM (low glucose; Gbico) supplemented with 10% FBS (Gbico) and 1% NEAA (Hyclone). Cells were changed to be cultured in DMEM without FBS overnight before use. Cell lysate for insulin signaling pathway detection was collected after stimulation with 100 nm insulin for 5-15 min.

### Animal assays

Male Hartley guinea pigs of about 8-weeks-old were housed in a controlled environment (12 h daylight cycle), with free access to food and water. Guinea pigs were fed with a chow diet or a HPD contained 15% palm oil (Animal center of Guangdong province). Serum samples were collected after 16 h of fasting. Biochemical parameters of serum were detected by automated analyzer (HITACHI-7020). Body composition was assessed by using a micro computed tomography (CT) scanner (HITACHI). IPGTT assays were performed after 16 h of fasting, and 20% D-glucose solution was injected intraperitoneally (i.p.) with a dose of 2 g/kg weight. ITT assays were performed after 4 h of fasting, and insulin was given i.p. with a dose of 1 IU/kg weight. Glucose content was detected by a glucometer (Roche). Liver and muscle samples for detection of insulin signal transduction were collected after stimulation of 2 IU/kg insulin for 20 min.

### Dosage information

Ascorbate (2-Phospho-L-ascorbic acid trisodium salt) was dissolved in water at different concentrations for oral intake ad libitum. The water was prepared freshly every day and guinea pigs administered ascorbate were kept alone in individual cages. Guinea pigs were supplied with 200 mg/kg or 600 mg/kg on average for low ascorbate (LA) and high ascorbate (HA) in reference of report described previously with modify [18]. The human equivalent dose is 36 mg/kg or 108 mg/kg referring to a conversion coefficient 0.18 between guinea pig and human according to body surface area. In consideration of molecular weight of sodium ascorbate we used (322.4 g/mol) and ascorbic acid (177 g/mol), there should be a conversion coefficient (0.55) for the same mole, which is 19.8 mg/kg or 59.4 mg/kg for ascorbate acid.

### Reagents

Sodium ascorbate was purchased from sigma (Shanghai, China). Intracellular TG and cholesterol content were detected by an enzyme kit (PPLYGEN, Beijing, China) as per the manufacturer’s detections. Periodic Acid Schiff (PAS) Staining Cells were stained with PAS to examine glycogen storage. Reagents were purchased from Qiangen (Hilden, Germany) and the assay was did as per users’ manuscript. Cell viability was detected by Cell Counting Kit-8 (Dojindo, Kumamoto, Japan) as per the manufacturer’s directions. Oil Red O solution, glucose and glycogen detection kits were all from Sigma (Shanghai, China). Antibodies against IR, phosphor-IR (Tyr^1150/1151^), AKT, phosphor-AKT (Thr^307^, Ser^473^), FOXO1, phosphor-FOXO1(Ser^256^), GSK3β, phospho-GSK3β(Ser^9^), β-tubulin were purchased from cell signaling technology (Danvers, Massachusetts, USA), β-actin was from Santa Cruz (Dallas, Texas, USA), SOCS3 was from Abcam (Cambridge, UK). Insulin ELISA kit for guinea pigs were from Jiancheng (Nanjin Jiancheng, Nanjin, China).

### Molecular cloning

Full length of SOCS3 was cloned from cDNA of HepG2 cells and inserted into a lentivirus vector, pSin. Virus packed in 293 T cells and supernatant contained active virus was collected for infection. Positive cells were screened out by puromycin resistance in a dose of 1.2 μg/ml for HepG2. Primers for PCR or shRNA sequence are submitted as Additional file 1.

### Metabolite extraction and lipidomics

Cell samples in chloroform/methanol/water (2/1/1, v/v/v) solution were vortexed for 1 min, and centrifuged at 3000 rpm for 10 min. Organic phage was collected and transferred to a new tube and lyophilized using nitrogen. Dried metabolites were reconstituted in 400 μl isopropanol/methanol (1/1,v/v) solution, vortexed, centrifuged at 12000 rpm for 10 min at 4 °C and supernatant was analyzed using LC-MS. A Kinetex C18 column (100 × 2.1 mm, 1.9 μm) and the following gradient: 0–2 min 30% mobile phase B; 2–20 min 100% B; 20–40 min 100% B; 40–40.01 min 30% B; 40.01–45 min 30% B, was applied for the experiment. Mobile phase A was acetonitrile/water (3:2, v/v), 10 mM ammonium formate. Mobile phase B was acetonitrile/ isopropanol (1:9, v/v), 10 mM ammonium formate and 0.1% formic acid. The flow rate was 0.4 ml/min, the column was at 45 °C. Parameters used for mass spectrometry were described as follows: positive ion mode, Heater Temp300 °C, Sheath Gas Flow rate, 45arb, Aux Gas Flow Rate, 15 arb, Sweep Gas Flow Rate, 1arb, spray voltage, 3.0KV, Capillary Temp, 350 °C, S-Lens RF Level, 30%. Scan ranges:200–1500; negative ion mode, Heater Temp300 °C, Sheath Gas Flow rate, 45arb, Aux Gas Flow Rate, 15arb, Sweep Gas Flow Rate, 1arb, spray voltage, 2.5KV, Capillary Temp, 350 °C, S-Lens RF Level, 60%. Scan ranges: 200–1500. Lipidomics assays was performed in Biocluster Inc.

### Statistical analysis

Statistical significance between two groups was assessed with an unpaired, two-sided Student’s *t* test using GraphPad Prism 6, and among three or more was assessed with one way ANOVA. All data represent means ± SEM. Statistical significance is denoted by *p < 0.05, **p < 0.01, and ***p < 0.001.

## Results

### Ascorbate ameliorates hepatic steatosis and insulin resistance in Guinea pigs

**Fig. 1:**
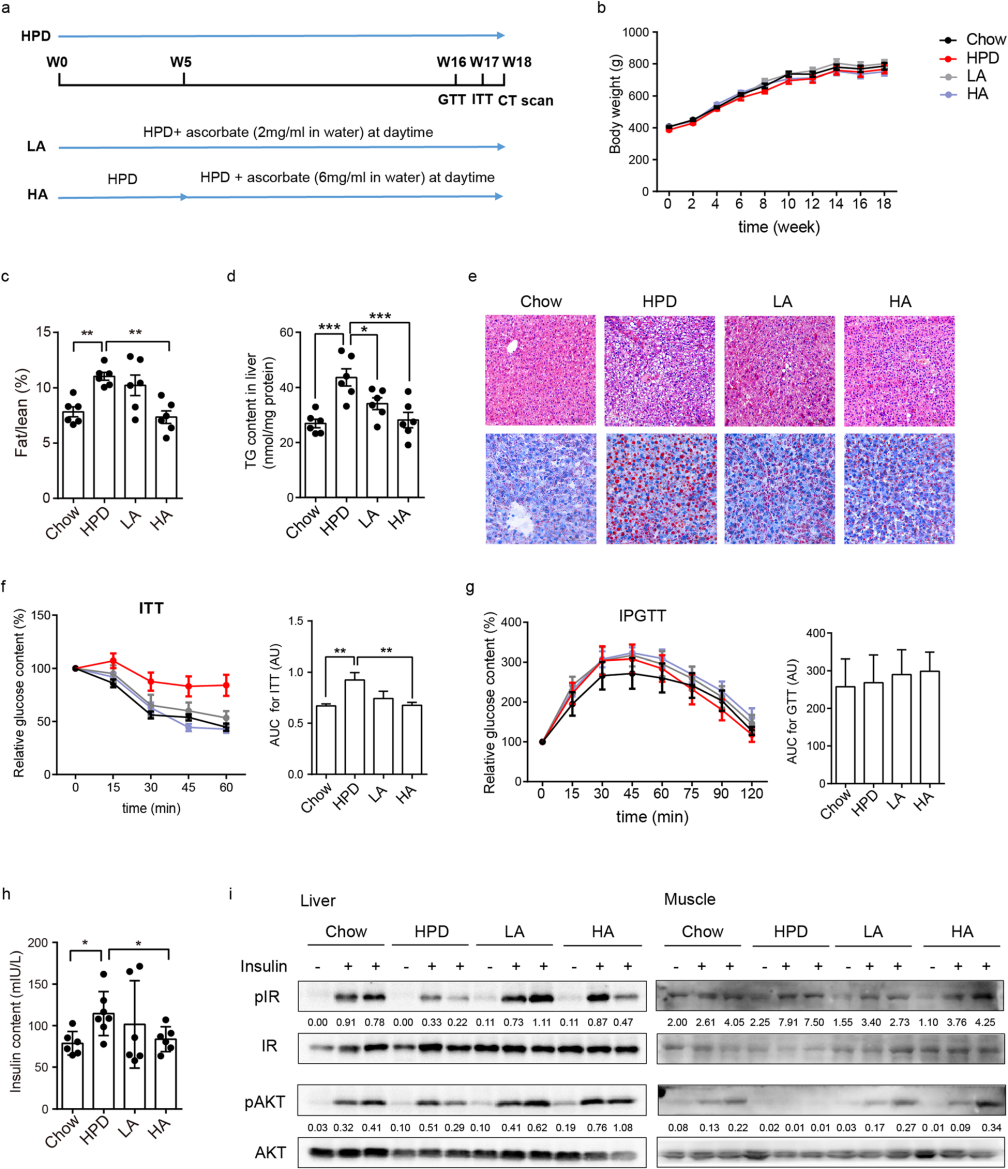
Ascorbate protects against hepatic steatosis and insulin resistance in guinea pigs. **a** Illustration of animal assay. **b** Body weight change within 18 weeks (*n* = 6). **c** Fat and lean ratio for guinea pigs (*n* ≥ 5). **d** TG contents in liver (*n* = 6). **e** Representative H&E stained and oil red O stained pictures of liver samples. **f** ITT assays performed on guinea pigs with/ without ascorbate intervention after fasting (*n* = 6). Area under curve (AUC) was quantified as depicted in colunm diagram. **g** IPGTT assays performed on guinea pigs with/ without ascorbate intervention after fasting (*n* = 6). Area under curve (AUC) was quantified as depicted in colunm diagram. **h** Insulin content in serum after fasting (*n* = 6). **i** Western blotting for insulin signaling pathway in liver (left) and muscle (right). Statistical significance was assessed with one way ANOVA

In order to establish an ideal animal model for testing ascorbate effects, the guinea pig was chosen as the candidate, since it is a species of rodent that cannot synthesis ascorbate like human kind and also is vulnerable to fat intake. Animals were fed with a chow diet or HPD respectively. Within 18 weeks, HPD elicited dyslipidemia, microvesicular steatosis and necrosis in liver gradually. To investigate whether ascorbate intervention could protect from metabolic disorder conditions, guinea pigs were orally administered different concentrations of ascorbate in different time intervals. The LA group was administered at the beginning and the HA group was administered after 5-weeks of feeding with HPD till the onset of steatosis (Fig. 1a).

Within the period of 18 weeks treatment, body weight showed no significant changes among groups (Fig. 1b). In line with this result, CT scan results showed ascorbate treatments had lower fat/lean ratio (Fig. 1c), suggesting ascorbate group animals got less fat during HPD lifestyle. Biochemical analysis confirmed that the ascorbate administrations decreased triglyceride (TG) content in both blood and liver tissue (Fig. 1d, Table 1). H&E and oil red O staining results showed that the ascorbate interventions ameliorated hepatic steatosis as well as lipid droplets accumulation, which was consistent with biochemical results (Fig. 1e). Sever metabolic syndrome and high dose of ascorbate absorption was related to liver and kidney damage. However, here in our animal model we found that index related to inflammation of liver and kidney showed no significantly difference, suggesting neither HPD nor ascorbate we used contributed to obvious damage (Additional file 1: Figure S1).

**Table 1:** Biochemical index in blood of guinea pig

	Chow (*n* = 6)	HPD(*n* = 6)	LA(*n* = 6)	HA(*n* = 6)
	content	content	content	content
TG (mM)	0.66 ± 0.053	0.97 ± 0.260	0.74 ± 0.196	0.66 ± 0.127
TC (mM)	2.26 ± 0.094	2.73 ± 0.250	2.93 ± 0.302	2.20 ± 0.561
HDL-C (mM)	0.19 ± 0.010	0.20 ± 0.019	0.25 ± 0.025	0.22 ± 0.032
LDL-C (mM)	2.09 ± 0.060	2.52 ± 0.188	2.63 ± 0.215	2.02 ± 0.536

* *TG* Triglyceride, *TC* Total cholesterol, *HDL-C* High density lipoprotein-cholesterol, *LDL-C* Low density lipoprotein-cholesterol

Indeed, ascorbate treatments improved the HPD impaired insulin sensitivity (Fig. 1f, h). To further investigate insulin signaling pathway in guinea pigs’ livers and skeletal muscle, we found that HPD impaired insulin signal transduction (Fig. 1i). However, ascorbate promoted cell sensitivity to insulin stimulation as phosphoryl levels of key proteins nearly returned to normal level. We also detected glucose tolerance in guinea pigs during the process and we found that the dietary style here in our experiment did not induce glucose tolerance as glucose consumption rate was no significant difference among groups (Fig. 1g). In animal research the intra-peritoneal glucose tolerance test (IPGTT) is used to assess the degree of diabetes. The differential responses between IPGTT and ITT most likely were caused by the pathological status and degrees of metabolism disorders.

### Ascorbate reduces lipid accumulation induced by PA in hepatic cells

**Fig. 2:**
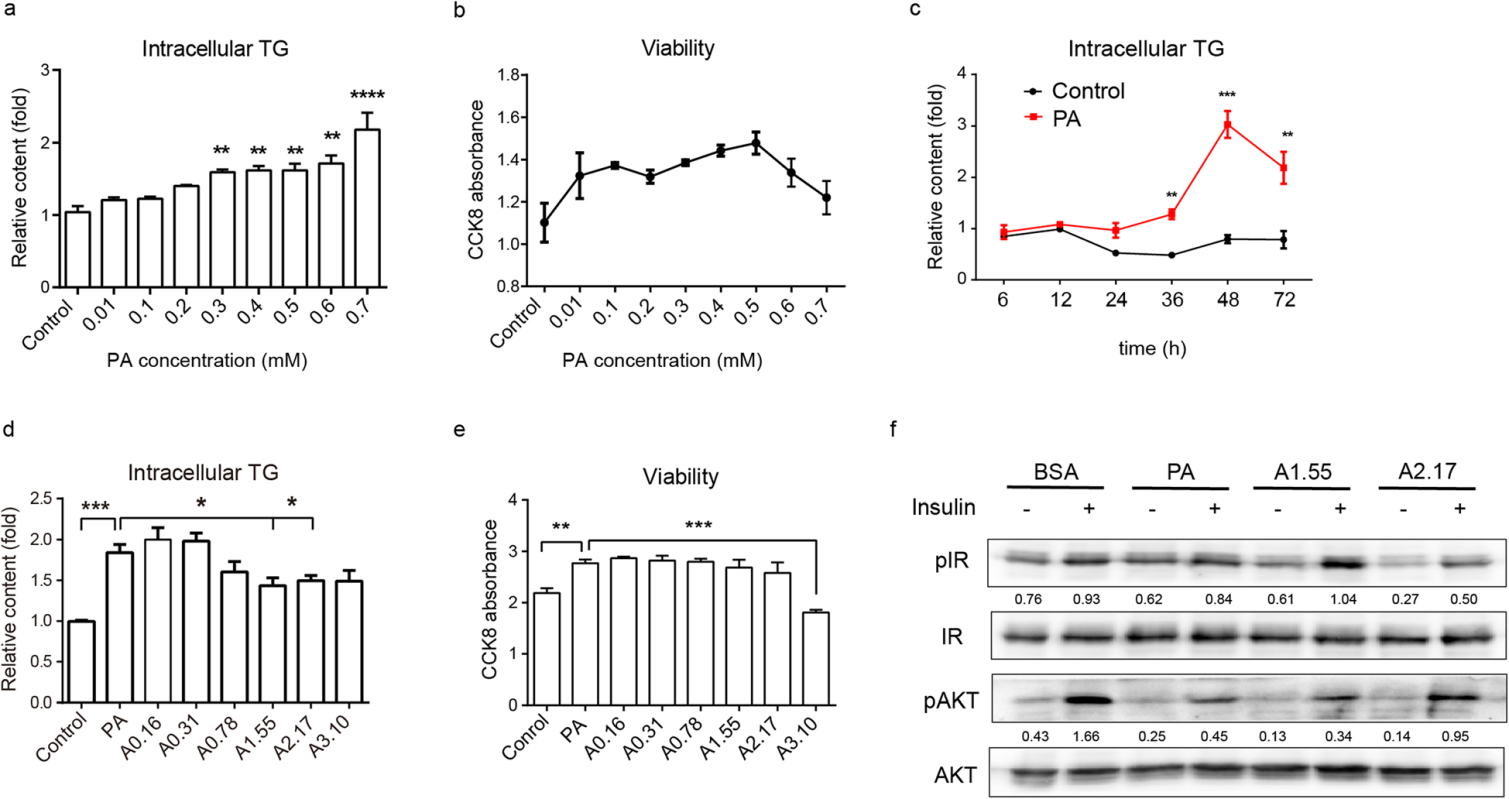
Ascorbate reduces lipid accumulaion and repairs insulin signal transduction in hepatic cells. With administration of various concentration of PA for 12 h, HepG2 cells were detected (**a**) intracellular TG content (*n* = 4) and (**b**) CCK8 absorbance for viability (*n* = 3) respectively. Significance was compared with control. HepG2 cells were treated with 0.5 mM PA for various interval, lysates of cells were collected to detect (**c**) intracellular TG content (n = 4). Significance was compared with PA group at each time point. HepG2 cells were co-treated with PA and ascorbate for 48 h, detected (**d**) intracellular TG content (*n* = 6) and (**e**) CCK8 absorbance for viability (*n* = 4). **f** Western blotting for phosphoryl level of proteins involved in insulin signaling pathway. Statistical significance was assessed with one way ANOVA

For further study, we then established the hepatic cellular steatosis model in HepG2 cell line that was illustrated by TG measurement and cell viability (Fig. 2a-c). As results above, intracellular lipid accumulation reached peak in HepG2 cells without obvious cell damage when cultured with 0.5 mM PA for 48 h.

In order to dissect the detail rescuing effects of ascorbate, we first analyzed the phenotypic outcomes on HpG2 cells treated with PA and different concentrations of ascorbate for 48 h. In the range of 1.55–2.17 mM, intracellular TG contents were significantly decreased while cell viability was not affected (Fig. 2d, e). The ascorbate rescuing effects were also confirmed on hepatocyte-like-cells derived from human embryonic stem cells H1, and the functions and maturation of these cells were also illustrated (Additional file 1: Figure S2A-D). The ascorbate dose curve assay showed that lower concentration level of ascorbate, initially at 0.31 mM, could ameliorate lipid accumulation by reducing TG content to a half compared to PA treatment alone (Additional file 1: Figure S2E). Consistent with that of liver tissue in guinea pigs, ascorbate also repaired the insulin signaling defects in vitro on the phosphoryl levels of IR and AKT in HepG2 cells (Fig. 2f).

### Ascorbate disrupts PA-induced hepatic lipid profiles and inhibits lipogenesis

**Fig. 3:**
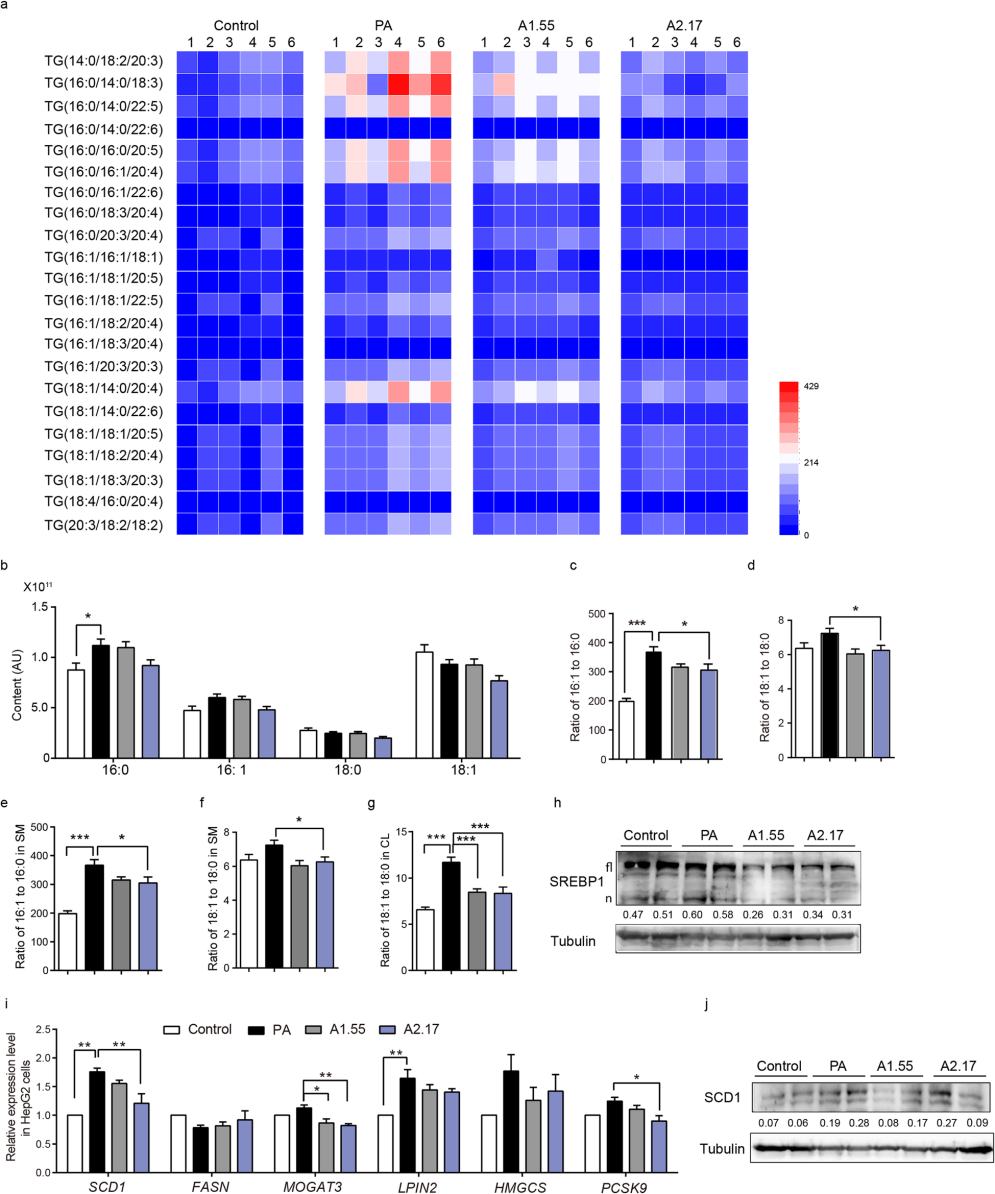
Ascorbate disrupts PA-induced hepatic lipid profiles and inhibits lipogenesis. LC/MS lipid profiling of HepG2 cells under PA exposure with two different ascorbate does for 48 h, *n* = 6. **a** Expression pattern of TG significantly changed among groups. **b** Intracellular palmitate (C16:0), palmitoleate (C16:1), stearate (C18:0) and oleic acid (C18:1) content.**c-g** Lipid desaturation indexes for total lipid species, cardiolipin (CL) and sphingomyelin (SM) respectively (16:1/16:0, 18:1/18:0). **h** Western blotting for SREBP1. **i** Relative expression level of lipogenesis related genes in HepG2 cells (n = 3). **j** Western blotting for SCD1. Statistical significance was assessed with one way ANOVA

Further, we performed liquid chromatography-tandem mass spectrometry (LC/MS) scanning assay for a global lipid profiling which showed that levels of saturated fatty acids and unsaturated fatty acids from TG were differentially enriched among groups (Fig. 3a). Ascorbate reduced palmitate (C16:0), palmitoleate (C16:1) and oleic acid (C18:1) levels, which resulted in decreased lipid desaturation index (16:1/16:0 and 18:1/18:0), mainly in cardiolipin (CL) and sphingomyelin (SM) (Fig. 3b-g). We then found that changes of lipid profiles was related to lipogenesis in cells. Sterol regulatory element binding proteins (SREBPs) are transcription factors which play a central role in lipid homeostasis. Both full-length (fl) and active SREBP1 (nSREBP1, n) content was decreased in ascorbate administration group (Fig. 3h). In line with this, the expression level of SREBP1 targeted genes were decreased, particularly *SCD1*, the enzyme essential for the first step of de novo lipogenesis, which contributes to lipid desaturation index, and showed expression pattern in a dose dependent manner (Fig. 3i, j, Additional file 1: Figure S2H). As gene information of guinea pigs wasn’t known completely, we mainly used hepatic cells for gene expression detection.

### Ascorbate repairs insulin signal transduction by inhibiting SOCS3 expression

**Fig. 4:**
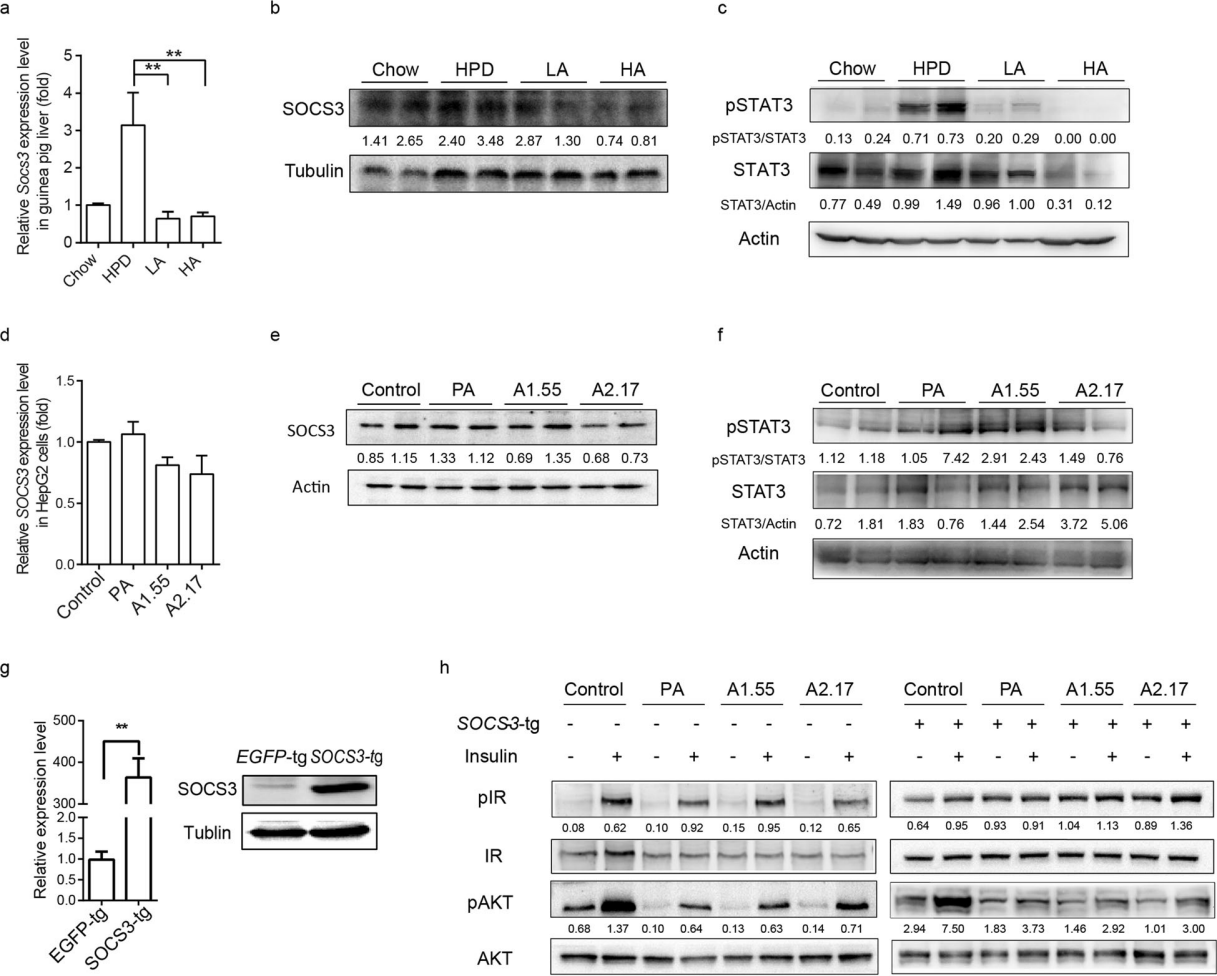
Ascorbate repairs insulin signal transduction by inhibiting SOCS3 expression. **a** Reltative *Socs3* mRNA expression level (*n* ≥ 5) and **b** SOCS3 protein content in liver of guinea pig. **c** Westen blotting for STAT3 and phosphrylational STAT3 (pSTAT3) in guinea pigs’ liver tissue. **d** Reltative *Socs3* mRNA expression level (n ≥ 5) and **e** SOCS3 protein content in HepG2 cells. **f** Westen blotting for Stat3 and phosphoryl STAT3 (pSTAT3) in HepG2 cells. Overexpressed *SOCS3* in HepG2 cells by lentivirus infection. **g** Relative mRNA expression level and protein content in cells. **h** Western blotting for phosphoryl level of insulin signaling pathway related key proteins. Statistical significance was assessed with one way ANOVA

SOCS3 is a repressor of insulin signaling pathway and it has been reported that inhibition of SOCS3 ameliorated hepatic steatosis and hypertriglyceridemia. We found that in our model, ascorbate reduced *Socs3* mRNA expression level as well as its protein content in liver tissue (Fig. 4a, b). Consistent with that of liver tissue in guinea pig, expression level of *SOCS3* in hepatic cells treated with ascorbate was decreased (Fig. 4d, e, Additional file 1: Figure S2 F, G). It seems ascorbate influenced the expression of *SOCS3* on transcriptional level. SOCS3 is a cytokine-inducible protein that can be elicited by IL6 through STATs [19, 20]. We then detected STAT3 content in liver tissue as well as hepatic cells, and found that its phosphorylation level was increased in HPD or PA group while it was reduced in ascorbate treated groups both in vivo and in vitro (Fig. 4c, f). It suggested that ascorbate inhibited SOCS3 through STAT3.

To further confirm whether the effect of ascorbate on repairing insulin signal transduction was related to *SOCS3*, we overexpressed *SOCS3* or *EGFP* in HepG2 cells (Fig. 4g). We then analyzed the insulin signaling pathway in cells. While overexpressing *SOCS3*, the ascorbate rescued phosphoryl level of IR and AKT was reduced (Fig. 4h), indicating that effects of ascorbate on insulin signal transduction was at least partially dependent on *SOCS3* expression.

### Overexpression of *SOCS3* compromise effects of ascorbate on lipogenesis

**Fig. 5:**
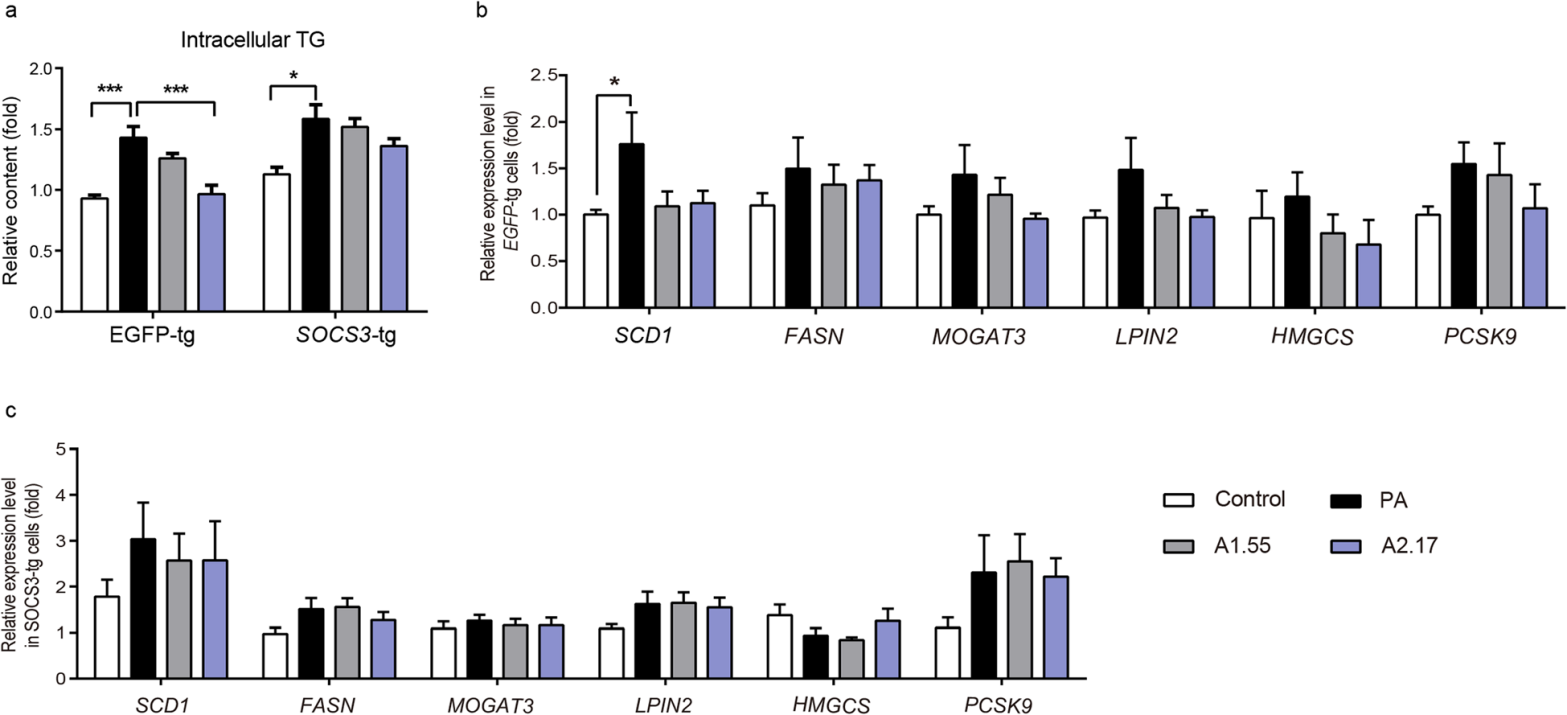
Overexpression of SOCS3 compromises effects of ascorbate on lipid accumulation. **a** Intracellular TG content of cells (n = 4). Expression level of lipogenesis related genes (**b**) in control or (**c**) *SOCS3*-tg cells (n = 3). Statistical significance was assessed with one way ANOVA

SOCS3 was reported to play a central role in metabolism and regulated expression of SREBP1c, which was an important transcriptional factor of lipid synthesis involved genes [21]. Measurement of the intracellular TG content indicated that the *EGFP* transgenic (*EGFP*-tg) cell line appeared ascorbate rescuing effects. However, the rescuing effects of ascorbate on lipid accumulation was abolished in the *SOCS3*-tg cell line (Fig. 5a). We described lipogenesis related genes were differentially changed before. We found that expression level of those genes was higher in *SOCS3*-tg cells compared with that in *EGFP*-tg cells, and regulation effects of ascorbate were compromised (Fig. 5b, c). This suggests that ascorbate reduced lipid accumulation at least partially through inhibiting *SOCS3* expression, and overexpression of *SOCS3* compromised this effect of ascorbate.

## Discussion

Steatosis refers to abnormal lipid accumulation in cells and the pathological consequence is cellular degeneration. It is reported PA exposure brings stress on lipid metabolism, resulting in abnormal lipogenesis, inflammation, and insulin resistance in multiple tissues and organs [22–24]. Here we use PA or diet with oil rich of PA (Additional file 1: Table S1) to conduct disease model in vitro and in vivo to mimic human lifestyle and find ascorbate ameliorates hepatic steatosis as well as insulin resistance. Collectively, we observed that ascorbate reduced lipid accumulation and improved insulin sensitivity by down-regulating *SOCS3* expression in vivo and in vitro.

SOCS3 is implicated in hypertriglyceridemia and functioning as a repressor of insulin signal transduction by inhibiting phosphorylation of IR as well as inducing degradation of IRS [25]. Inhibition of *SOCS3* ameliorates hepatic steatosis and hypertriglyceridemia, while knockout of it in liver enhances lipogenesis in obesogenic milieu in mice as there was increased hypothalamic SOCS3 [26]. Here we showed ascorbate partially inhibited SOCS3 expression and ameliorated hepatic steatosis. SREBP1 preferentially enters nucleus to enhance transcription of genes required for lipogenesis when activated by proteolytic cleavage [27]. Our in vitro assays showed ascorbate reduced SREBP1 content, lipid metabolites related genes, especially *SCD1*, which initiates lipogenesis by catalyzing the conversion of C16:0 and C18:0 to C16:1 and C18:1, and lipid desaturation index. As it was reported that SOCS3 was involved in regulation of SREBP1. We believe ascorbate reprogramed lipid profiles via inhibiting lipogenesis through SOCS3. Alterations in lipid composition can influence biological activity such as material exchange as well as cellular signal transduction [28, 29]. The biological meaning of the altered fingerprinting of lipids under the ascorbate administration is worth to be further investigated. SOCS3 is also a leptin resistance inducer, thus leads to increased lipogenesis, obesity and hepatic steatosis [30, 31]. In our animal model, we found no obvious significant difference in leptin content among groups, suggesting there was no leptin resistance.

We pursue the mechanism of ascorbate regulates *SOCS3* expression is in relevance to transcriptional regulation. Ascorbate promotes the activity of Tet enzymes and Tet2 is reported to recruit Hdac2 to specifically repress IL-6, which is a well-known inducer of SOCSs family through JAK-STAT signaling [32–35]. However, intracellular and extracellular IL6 contents were rarely detected, we wonder even the regulation activity of ascorbate handling the expression pattern of *SOCS3* through IL6. If ascorbate may act as an enzymatic cofactor, there is a tendency that ascorbate or its metabolites might work with protein kinase involved STAT3 signaling, which should further be investigated.

Ascorbate is well tolerance. Compared to the NIH suggestions that the oral RDA of ascorbate is 75–90 mg a day for a health adult, our animal experiments supplied the ascorbate in drink water with higher dose which has been tested in other published work and proven that those dose region has no toxic effects for guinea pigs. In our experiments, the high dose of ascorbate has weak effects on pH value, and the administered guinea pigs had no damage on kidneys and livers (Additional file 1: Figure S1). As limited of bioavailability, only about 20–30% of ascorbate supplied could be available for utilization in usual when gavage, and intravenous injection (i.v.) might administer lower dose of it and reduce risks for some side effects. The safety of ascorbate has been confirmed in clinical trials which up to 10 mM via i.v. for human [36]. We assume our data may provide a reference for clinical use of ascorbate on metabolic syndromes.

In summary, we find a novel insight that ascorbate prevents hepatic steatosis by inhibiting *SOCS3* and improving insulin sensitivity in vitro and in vivo. These findings not only illustrate the cellular basis that ascorbate supplement is beneficial for clinical metabolic syndromes, but also provides a clue to generate reporter cell lines with monitored key target proteins functions for drug screening of metabolic syndromes.

## Supplementary information

**Additional file 1: Figure S1.** Ascorbate administration did not cause obvious damage in vivo and in vitro. **Figure S2.** Ascorbate ameliorates lipid accumulation in hepatocyte-like cells. **Table S1.** Composition of palm oil for diet. **Table S2.** RT-qPCR primers. **Table S3.** PCR primers.

## Data Availability

The datasets used and/or analysed during the current study are available from the corresponding author on reasonable request.

## Abbreviations

FOXO1: Forkhead box protein O1
GSK3β: Glycogen synthase kinase 3β
HPD: High pail oil diet
ICG: Indocyanine green
IR: Insulin receptor
LC/MS: Liquid chromatography–mass
NAFLD: Nonalcoholic fatty liver disease
PA: Palmitic acid
PAF: Platelet-activating factor
PC: Phosphatidylcholine
PE: Phosphatidylethanolamine
SM: Sphingomyelin
SOCS3: Suppressor of cytokine signaling 3
TG: Triglyceride
