# Supplementary material for: Ascorbate protects liver from metabolic disorder through inhibition of lipogenesis and suppressor of cytokine signaling 3 (SOCS3)
Source: Nutr Metab (Lond). 2020 Mar 4;17:17. doi: 10.1186/s12986-020-0431-y (PMC7057613; doi:10.1186/s12986-020-0431-y)
Supplement: Supplementary file 1 — Additional file 1: Figure S1. Ascorbate administration did not cause obvious damage in vivo and in vitro. Figure S2. Ascorbate ameliorates lipid accumulation in hepatocyte-like cells. Table S1. Composition of palm oil for diet. Table S2. RT-qPCR primers. Table S3. PCR primers. [file 12986_2020_431_MOESM1_ESM.docx]

**Additional file 1**

**Cell culture**

Human embryonic stem cells, H1, were cultured in Stem Cell Medium (Propertech) during proliferation stage. During differentiation, cells were changed to medium containing B27 insulin minus following the sequence, 100ng/ml Activin A and 3μM CHIR99021 at day 1, Activin A at day 2-3, 20ng/ml BMP2, 20ng/ml BMP4 and 30ng/ml FGF4 at day 4-8, 20ng/ml HGF and 20ng/ml KGF at day 9-13, 10ng/ml OSM and 0.1μM Dexamethasone at d14-18, hepatocyte culture medium (HCM, Lonza) EGF minus thereafter.

**Reagents**

For immunofluorescence, antibodies against Albumin were from Gene Tex (Irvine, California, USA), E-cadherin, CK18, and CYP3A4 were from R&D (Minneapolis, Minnesota, USA).

**
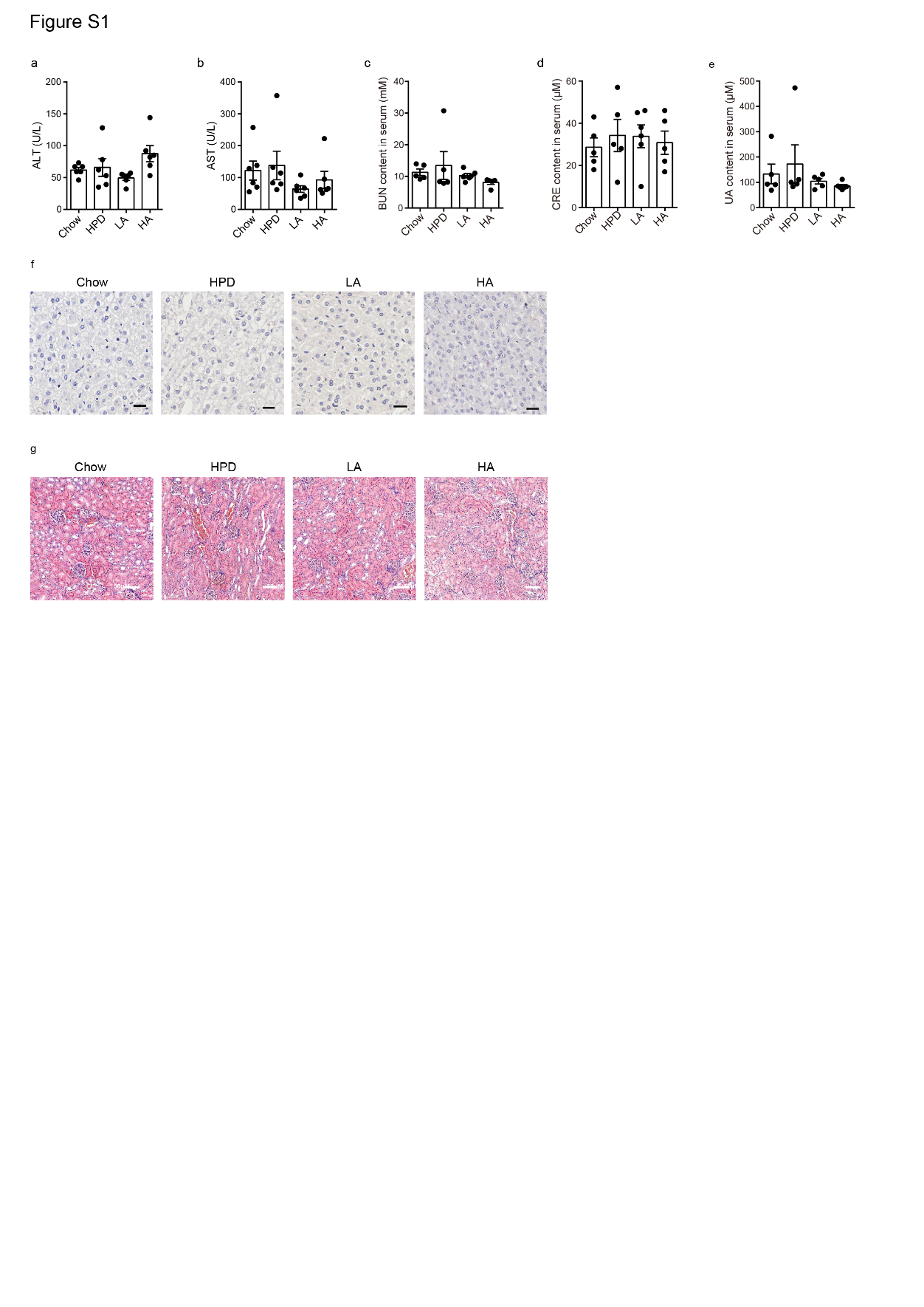
**

**Fig. S1. Ascorbate administration did not cause obvious damage *in vivo* and *in vitro*.**

Liver and kidney functions were detected by serum analysis at week 18, (**a**) alanine aminotransferase (ALT) content, (**b**) aspartate aminotransferase (AST) content, (**c**) blood urea nitrogen (BUN) content, (**d**) creatinine (CRE) content, (**e**) uric acid (UA) content in serum (n≥5). (**f**) Representative TUNEL and (**g**) H&E staining, scale bar, 20μm. Statistical significance was assessed with one way ANOVA.


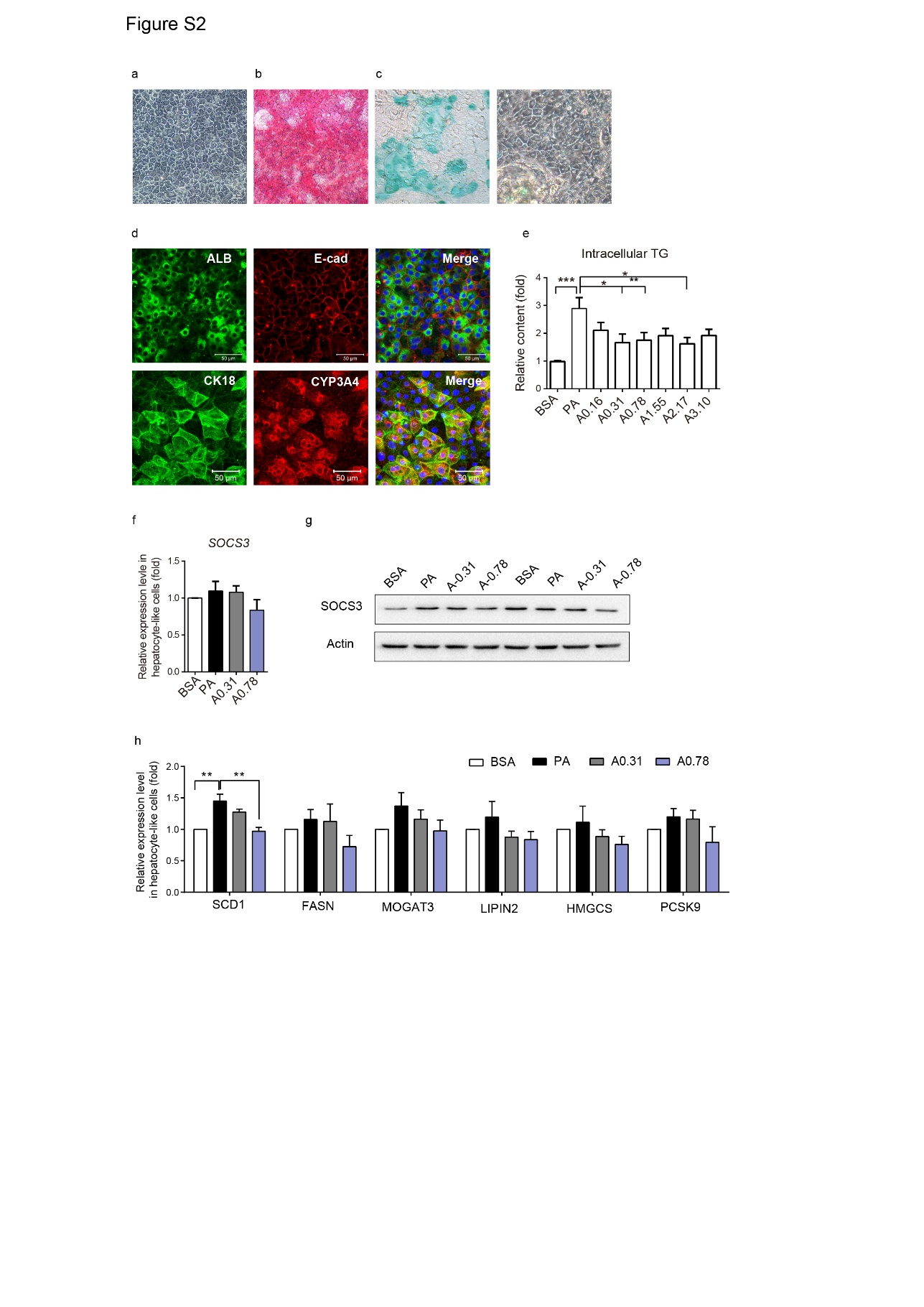


**Fig. S2.** **Ascorbate ameliorates lipid accumulation in hepatocyte-like cells**

Hepatocytes-like-cells were cultured for 21 days till maturation before hepatocytes were characterized. (**a**) Morphology of hepatocyte-like-cells. (**b**) PAS staining for glycogen storage. (**c**) ICG up taking (left) and releasing (right) assays. (**d**) Immunofluorescence staining for ALB (green), E-cadherin (red), CK18 (green) and CYP3A4 (red). Hepatocyte-like-cells differentiated from human embryonic stem cell line H1 were co-treated with PA and ascorbate for 48h. (**e**) Intracellular TG (n=3). (**f**) Rletative *SOCS3* mRNA expression level (n=3) and (**g**) protein content. (**h**) Relative expression level of lipogenesis related genes (n=3). Statistical significance was assessed with one way ANOVA.

**Supplementary table**

**Table S1 Composition of palm oil for diet**

| **Test item(s)** | **Test results** | **Unit** |
| --- | --- | --- |
| Caprylic Acid (C8:0) | 0.02 | g/100g |
| Capric Acid (C10:0) | 0.02 | g/100g |
| Lauric Acid (C12:0) | 0.18 | g/100g |
| Myristic Acid (C14:0) | 0.98 | g/100g |
| Pentadecanoic Acid (C15:0) | 0.05 | g/100g |
| Palmitic Acid (C16:0) | 44.86 | g/100g |
| Heptadecanoic Acid (C17:0) | 0.15 | g/100g |
| Stearic Acid (C18:0) | 4.3 | g/100g |
| Arachidic Acid (C20:0) | 0.34 | g/100g |
| Behenic Acid (C22:0) | 0.05 | g/100g |
| Lignoceric Acid (C24:0) | 0.06 | g/100g |
| Palmitoleic Acid (C16:1-9c) | 0.12 | g/100g |
| cis-9-Oleic Acid (C18:1-9c) | 30.75 | g/100g |
| Petroselaidic Olaidic  Transvaccenic (C18:1[trans-6].[trans-9],[trans-11]) | 0.09 | g/100g |
| Linleic Acid (C18:2-9c,12c) | 7.58 | g/100g |
| α-Linolenic Acid  (C18:3(ALA)-9c,12c,15c) | 0.11 | g/100g |
| Trans-12,cis-9-Linoleic Acid  (C18:2 trans-12,cis-9) | 0.15 | g/100g |
| Trans-9,cis-12-Linoleic Acid  (C18:2trans-9,cis-12) | 0.15 | g/100g |

**Table S2 RT-qPCR primers**

| Name | Sequence (5’-3’) |
| --- | --- |
| Human *βACTIN* forward | CTCCTCCTGAGCGCAAGTACTC |
| Human *βACTIN* reverse | TCCTGCTTGCTGATCCACATC |
| Human *MOGAT3* forward | CCAACCACTTCCAAAACCTTGC |
| Human *MOGAT3* reverse | TGCCCGGTTCCTTATCCACT |
| Human *LPIN* forward | GCAGTGCAGTGGATCTTCACA |
| Human *LPIN* reverse | GTGAGGTGGCAAGGTAAGCA |
| Human *HMGCS* forward | GATGTGGGAATTGTTGCCCTT |
| Human *HMGCS* reverse | ATTGTCTCTGTTCCAACTTCCAG |
| Human *PCSK9* forward | CTGTATGCTGGTGTCTAGGAGA |
| Human *PCSK9* reverse | CTGTATGCTGGTGTCTAGGAGA |
| Human *SCD1* forward | TTCCTACCTGCAAGTTCTACACC |
| Human *SCD1* reverse | CCGAGCTTTGTAAGAGCGGT |
| Human *SOCS3* forward | CAGCTCCAAGAGCGAGTACC |
| Human *SOCS3* reverse | TGTCGCGGATCAGAAAGGTG |
| Guinea pig *βActin* forward | CGTTACCAACTGGGACGACA |
| Guinea pig *βActin* reverse | CATCTTCTCACGGTTGGCCT |
| Guinea pig *Socs3* forward | CTACTGGAGTGCCGTAACCG |
| Guinea pig *Socs3* reverse | GATGCGCAGGTTCTTTGTCC |

**Table S3 PCR primers**

| Name | Sequence (5’-3’) |
| --- | --- |
| *SOCS3* sense primer | ATGGTCACCCACAGCAAGTTTC |
| *SOCS3* antisense primer | TTAAAGCGGGGCATCGTACTG |
| *SOCS3* promoter sense primer | CCCTTTCAGCACCTCATTATCC |
| *SOCS3* promoter antisense primer | GCGCTCCTCCTTCCTACCTG |
